# Awareness of mpox-related knowledge among men who have sex with men in China

**DOI:** 10.1186/s12889-023-15503-3

**Published:** 2023-03-30

**Authors:** Min Zheng, Wenyan Chen, Xiaohan Qian, Rui Tao, Lin Ma, Feng Zhou, Zhilin Zhu, Yongming Yao, Guanghong Yang

**Affiliations:** 1 Guizhou Provincial Center for Disease Control and Prevention, Guiyang, 550004 Guizhou China; 2 School of Public Health, the Key Laboratory of Environmental Pollution Monitoring and Disease Control, Ministry of Education, Guizhou Medical University, Guiyang, 550025 Guizhou China

**Keywords:** Mpox, Men who have sex with men, China, Knowledge

## Abstract

**Background:**

With the rapid spread of the mpox epidemic, cases have emerged in multiple countries, mainly among men who have sex with men. Because of the connectedness of today’s world, countries have to be prepared to face risks in advance. Therefore, this study aimed to investigate awareness of mpox-related knowledge among men who have sex with men in China.

**Methods:**

With the assistance of the social organizations of men who have sex with men, a cross-sectional survey of men who have sex with men in China was conducted through an online questionnaire between July 1 and July 18, 2022. A nationwide sample of Chinese men who have sex with men (N = 3,257) was recruited.

**Results:**

Only 36.9% of participants had mpox-related knowledge. Awareness of mpox-related knowledge among respondents was positively associated with those in older age groups (33 to 42 years and 51 years or older) (adjusted odds ratio [AOR] = 1.31; 95% confidence interval [CI]: 1.03–1.67, AOR = 1.61; 95% CI: 1.16–2.24; respectively), married (AOR = 1.55; 95% CI: 1.09–2.19), and those with a graduate degree or above (AOR = 2.14; 95% CI: 1.11–4.13), while negatively associated with those living in the western parts of China (AOR = 0.74; 95% CI: 0.60–0.92), and those who were unsure of their history of Human Immunodeficiency Virus (HIV) status (AOR = 0.44; 95% CI: 0.30–0.63).

**Conclusion:**

Mpox-related knowledge is fairly low among men who have sex with men in China. China needs to spread knowledge to the public through multiple channels, especially in key populations (men who have sex with men, HIV-infected, etc.), and take preventive measures to effectively avoid outbreaks of mpox.

**Supplementary Information:**

The online version contains supplementary material available at10.1186/s12889-023-15503-3

## Background

From January 1 through September 4, 2022, 52,996 cases of mpox, including 18 deaths, were reported by the World Health Organization (WHO) [1]. This epidemic, first confirmed in May 2022 in the United Kingdom (UK), has spread globally from Europe to North America, Africa, Australia, and Asia [2, 3]. It was announced as a “public health emergency of international concern by the WHO on [July 23] 2022” [4]. As of September 16, 2022, there have been five cases of mpox in China, including three in Taiwan province, one in Hongkong city, and one in Chongqing [5, 6].

Mpox, presently a zoonotic disease, is caused by the mpox virus (MPXV) which belongs to the Orthopoxvirus genus of the Poxviridae family [7]. Since first being reported in humans in 1970, mpox has become endemic in the Democratic Republic of the Congo (DRC) [8]. There are two distinct genetic clades of MPXV: the Central African (Congo Basin) clade and the West African clade [9]. The primary infection sources for mpox are rodents and primates infected with MPXV, which include monkeys, chimpanzees, and humans [10]. Since 2003, importation- and travel-related cases of mpox have spread outside Africa [11]. MPXV invades the body through body fluids, mucous membranes, and broken skin [12, 13]. Human-to-human transmission principally occurs via close or indirect contact with contaminated items [14]. Those infected with mpox mainly present with fever, headache, lymphadenopathy, and rash on the face and genitals [15, 16]. Since May 2022, the number of reported cases of mpox has grown, mostly affecting men who have sex with men [17]. In this outbreak, close contact during sexual or intimate activities is a route of transmission among men who have sex with men who have multiple sexual partners or practice condomless anal intercourse [18, 19]. It may be the main driver behind the rise in cases worldwide [20]. Smallpox vaccination imparts some degree of cross-protection against MPXV, with a preventive effect of approximately 85% [21]. Nevertheless, the global smallpox vaccination program ended after 1980 [22]. Another explanation for the increase in mpox outbreaks may be related to the population’s declining immunity to smallpox over time [23]. A study based on data from four clusters-Italy, Australia, Portugal, and the UK-revealed health risk and behavioral factors for mpox infection, including being a male, having sex with other men, having condomless anal intercourse, having multiple sexual partners, people living with human immunodeficiency virus (HIV) and a history of sexually transmitted infections (STIs) (including syphilis) [24, 25].

At present, the ongoing epidemic of viral infection in humans differs from preceding outbreaks in terms of sources of infection, routes of transmission, vulnerable populations, sexual identity, and risky behaviors. China has a large population, and men who have sex with men living in China are a hardly reached population [26, 27]. Bisexuality and commercial sex are also frequently observed in men who have sex with men in China [28]. Therefore, it is urgent for men who have sex with men, a key population, to acquire knowledge related to mpox to protect themselves. The results of an online study that surveyed men who have sex with men living in the Netherlands showed that 52% of respondents paid high attention to mpox, but lacked knowledge and understanding of the ongoing epidemic of mpox. Men who have sex with men in the Netherlands realized that the increase in the number of sexual partners and activities may have a higher risk of mpox, but they did not know that HIV infection may be a risk factor for mpox [29]. Mastering disease-related knowledge is a significant basis for cultivating positive attitudes and further promoting behavior change [30]. It is important to acknowledge that knowledge is not certain to change behavior, but raising public awareness during the public health crisis can significantly increase the adoption of protective health behaviors and thus curb the spread of infectious diseases [31, 32]. However, research on awareness of mpox-related knowledge among men who have sex with men in China is still somewhat rare. This study aims to explore mpox-related knowledge and associated factors among men who have sex with men in China.

## Methods

### Study population and design

Data for our study were drawn from a cross-sectional online questionnaire of men who have sex with men in China between July 1 and July 18, 2022. A total of 3,257 respondents were surveyed in this study, primarily through convenience sampling. The link to this survey was distributed to members of the men who have sex with men community, who were also asked to share it with other members in their communities. Enrollment was limited to males who (1) were aged 16 and older; (2) identified as gay or bisexual; (3) self-reported having had sex with at least one man in the past 12 months; and (4) provided informed consent. Participation in the survey was voluntary. The study’s design, details, and procedures were approved by the Ethics Committee of the Guizhou Center for Disease Control and Prevention (Q 2022-02).

### Measures

The questionnaire of our survey was designed on the basis of prior literature [33]. Experts in related fields were invited to check its content and structure. Eligible participants were asked about their current age (≤ 22, 23–32, 33–42, ≥ 43), province, racial/ethnic identity, marital status (married, unmarried, divorced/widowed), employment status (farmer, student, employee of an enterprise or public institution, worker, businessman, commercial sex worker, unemployed, other), educational level (junior high school and below, high school/technical secondary school, junior college, undergraduate school, master’s degree or above), sexual orientation (gay, bisexual), history of HIV, history of syphilis, male sex partners in the past month, and condom use.

The outcome variable in this study was knowledge about mpox. Based on the principles of infectious disease prevention and control, we defined knowledge of mpox in terms of sources of infection, transmission routes, susceptible populations, prevention and treatment. We used the following five questions about mpox-related knowledge:

1. What are the sources of mpox infection?
2. What are possible transmission routes?
3. Which groups are susceptible to mpox?
4. How can one avoid contracting mpox?
5. Is there a specific treatment/drug for viral infection?

Questions 1–4 are all multiple-choice questions containing correct answer and a “Don’t Know” option. There were no incorrect options. Each question was considered correct if at least 1 right option is chosen. However, question 5 was a single-choice item with only one correct option and “Don’t Know” option. If the right option was selected, the question was considered correct. Those who answered all five questions correctly were considered to have mpox-related knowledge, otherwise they were considered not to have this knowledge. The full list of possible answers to the above 5 knowledge questions related to mpox, and the results of the answers can be found in the supplementary Table 1.

### Statistical analyses

We removed invalid data from all questionnaires. Data from the 3257 questionnaires were analyzed using SPSS (version 22.0) and Prism version 8. Categorical data are presented as frequencies and percentages. For comparison of different groups for categorical variables, the Pearson chi-square test was performed. Variables whose *P* value was < 0.2 in the univariate analyses were included in the multivariable analysis. Multivariable binary logistic regression was carried out to estimate the adjusted odds ratio (AOR) with its 95% confidence intervals (CIs) to assess the factors for mpox-related knowledge. *P* < 0.05 using two-sided tests was considered statistically significant.

## Result

A total of 4,792 questionnaires were collected, and 3,257 participants were eligible for the survey. We retained a sample of 3,257 after eliminating those who did not meet the inclusion criteria.

### Sociodemographic traits and risky behaviors

**Fig. 1:**
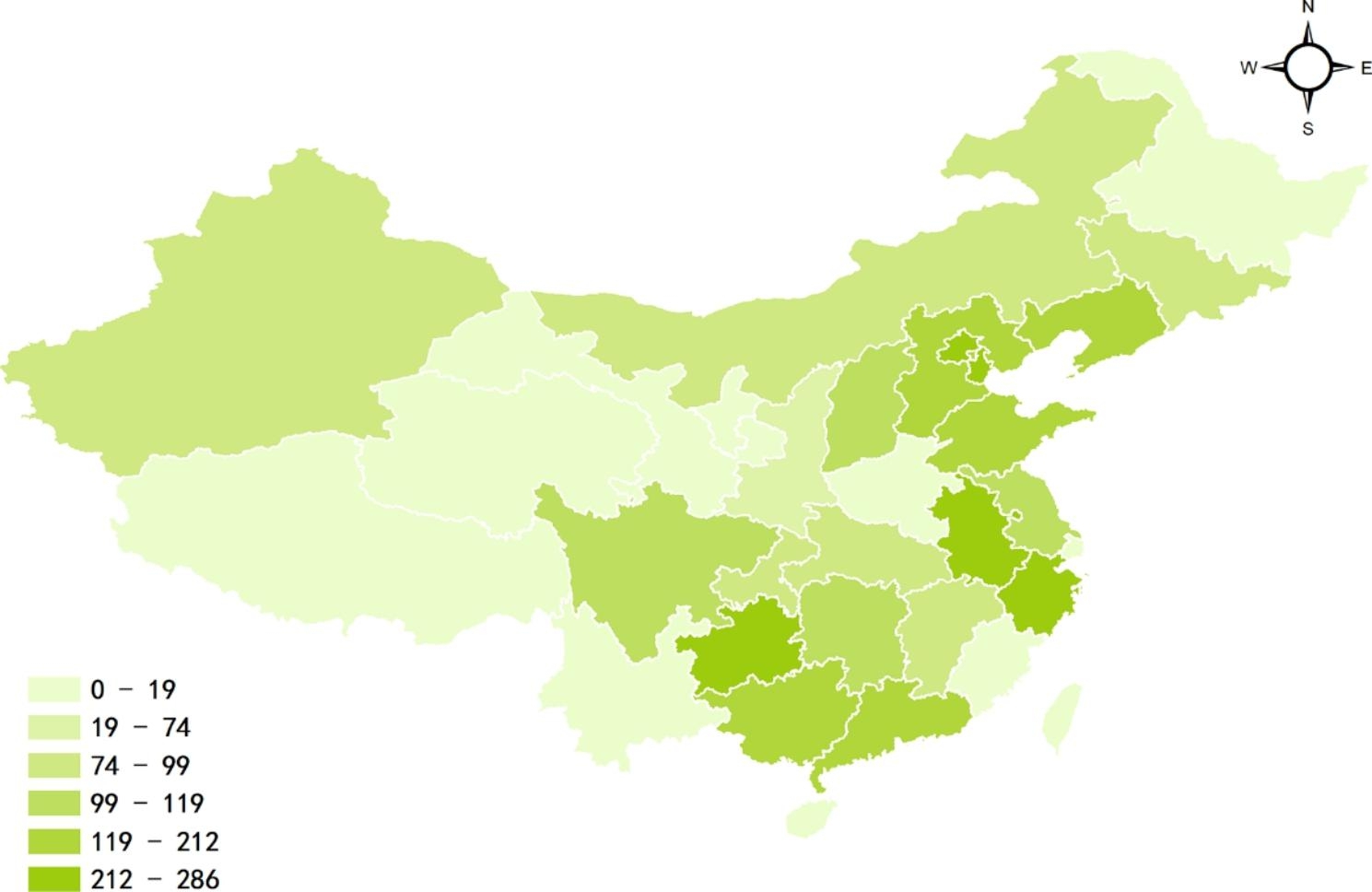
Distribution of study participants in China (N = 3,257)

Figure 1; Table 1 present sociodemographic traits and risky behaviors among Chinese men who have sex with men. The participants in this survey were distributed across 28 provinces (Fig. 1). The median age of the sample was 29 years old (interquartile range: 24–36). A total of 46.2% (n = 1,505) were between 23 and 32 years old. Compared to the distribution of the Chinese general population, the respondents in this survey tended to be younger. More than half of the participants (59.3%) lived in the eastern region, followed by the central (25.5%) and western (15.3%) regions. In terms of ethnic background, 92.1% were Han and 7.9% were minorities. The majority (80.8%) had never been married and had an education at the college/undergraduate level (65.4%) (Table 1).

**Table 1:** Demographic traits, history of STIs, and risky behaviors of MSM in China (N = 3,257)

Characteristics	N (%)	Mpox-related knowledge		*χ* ^*2*^	*P*
		Not Knowledgeable	Knowledgeable		
**Socio-demographics**					
**Age group**				5.182	0.159
≤ 22	544(16.7)	362(17.6)	182(15.1)		
23–32	1505(46.2)	955(46.5)	550(45.7)		
33–42	849(26.1)	516(25.1)	333(27.7)		
≥ 43	359(11.0)	221(10.8)	138(11.5)		
**Region**				5.207	0.074
Eastern	1930(59.3)	1200(58.4)	730(60.7)		
Central	830(25.5)	518(25.2)	312(25.9)		
Western	497(15.3)	336(16.4)	161(13.4)		
**Ethnic group**				1.985	0.159
Han	3001(92.1)	1903(92.6)	1098(91.3)		
Minority group	256(7.9)	151(7.4)	105(8.7)		
**Marital status**				7.426	0.024*
Married	408(12.5)	277(13.5)	131(10.9)		
Unmarried	2632(80.8)	1653(80.5)	979(81.4)		
Divorced/widowed	217(6.7)	124(6.0)	93(7.7)		
**Occupation**				23.248	0.002*
Farmer	148(4.5)	110(5.4)	38(3.2)		
Student	618(19.0)	401(19.5)	217(18.0)		
Employee of enterprise or public institution	902(27.7)	522(25.4)	380(31.6)		
Worker	342(10.5)	222(10.8)	120(10.0)		
Businessman	282(8.7)	190(9.3)	92(7.6)		
Commercial sex worker	502(15.4)	309(15.0)	193(16.0)		
Unemployed	295(9.1)	192(9.3)	103(8.6)		
Other	168(5.2)	108(5.3)	60(5.0)		
**Educational background**				54.468	< 0.001*
Illiteracy and primary school	50(1.5)	35(1.7)	15(1.2)		
Junior high school	254(7.8)	202(9.8)	52(4.4)		
High school/ Technical secondary school	525(16.1)	358(17.4)	167(13.9)		
Junior college/ Undergraduate school	2130(65.4)	1303(63.5)	827(68.7)		
Master’s or above	298(9.2)	156(7.6)	142(11.8)		
**Sexual identity**				0.059	0.808
Gay	2619(80.4)	1649(80.3)	970(80.6)		
Bisexual	638(19.6)	405(19.7)	233(19.4)		
**History of HIV**				24.433	< 0.001*
Yes	867(26.6)	520(25.3)	347(28.8)		
No	2189(67.2)	1376(67.0)	813(67.6)		
Unsure	201(6.2)	158(7.7)	43(3.6)		
**History of syphilis**				10.169	0.006*
Yes	475(14.6)	298(14.5)	177(14.7)		
No	2658(81.6)	1661(80.9)	997(82.9)		
Unsure	124(3.8)	95(4.6)	29(2.4)		
**Male partners in the past month**				0.583	0.747
0	1433(44.0)	913(44.4)	520(43.2)		
1–2	1569(48.2)	979(47.7)	590(49.1)		
≥ 3	255(7.8)	162(7.9)	93(7.7)		
**Condom use**				2.967	0.227
Never	146(4.5)	100(4.9)	46(3.8)		
Sometimes	891(27.3)	572(27.8)	319(26.5)		
Every time	2220(68.2)	1382(67.3)	838(69.7)		

^*^P < 0.05

Most participants self-identified as gay (80.4%) and bisexual (19.6%), while a minority reported a history of HIV (26.6%), and 14.6% reported a history of syphilis. A total of 48.2% had had 1–2 sexual partners in the past month. The majority (68.2%) had used a condom with their sexual partner every time, while 27.3% reported sometimes using condoms when having sex with a male sexual partner (Table 1).

### Awareness of mpox-related knowledge

In the sample, 36.9% of respondents had awareness of mpox-related knowledge, while 63.1% did not. Compared to the latter group, males who had mpox-related knowledge and males who had not. Males who had mpox-related knowledge had significant differences in marital status (*p* = 0.024), occupation (*p* = 0.002), and education level (*p* < 0.001). As Table 1 shows, a history of HIV and a history of syphilis are potential influential factors in having knowledge of mpox in the men who have sex with men population.

### Factors associated with mpox-related knowledge

To identify the factors influencing mpox knowledge, we conducted logistic regression analyses. Age was associated with mpox-related knowledge in that men who have sex with men in older age groups (33–42 and 51+, AOR = 1.31; 95% CI: 1.03–1.67; p = 0.030) were more likely to have mpox-related knowledge than those ≤ 22 years old (AOR = 1.61; 95% CI: 1.16–2.24; p = 0.004). Having mpox-related knowledge was also more frequent among divorced or widowed men who have sex with men than among those who were married (AOR = 1.55; 95% CI: 1.09–2.19; *p* = 0.014). Participants living in western parts of China were less likely to have mpox-related knowledge than those living in the east (AOR = 0.74; 95% CI: 0.60–0.92; *p* = 0.007). Men who have sex with men with a graduate degree or above had more mpox-related knowledge than those who were illiterate or had a primary education level only (AOR = 2.14; 95% CI: 1.11–4.13; *p* = 0.023). Males who were unsure of their HIV status were less likely to have mpox-related knowledge than those who were living with HIV (AOR = 0.44; 95% CI: 0.30–0.63; *p* < 0.001) (Table 2).

**Table 2:** Factors associated with mpox-related knowledge (logistic regression, N = 3,257)

Variables	AOR	95% CI	*P*
Age group			
≦ 22	1(ref)		
23–32	1.05	0.85–1.30	0.652
33–42	1.31	1.03–1.67	0.030*
≥ 43	1.61	1.16–2.24	0.004*
**Marital status**			
Married	1(ref)		
Unmarried	1.27	0.98–1.64	0.077
Divorced/widowed	1.55	1.09–2.19	0.014*
**Region**			
Eastern	1(ref)		
Central	0.93	0.78–1.10	0.392
Western	0.74	0.60–0.92	0.007*
**Education background**			
Illiteracy and primary school	1(ref)		
Junior high school	0.53	0.27–1.05	0.069
High school/Technical secondary school	1.07	0.56–2.02	0.845
Junior college			
Undergraduate school	1.52	0.82–2.83	0.186
Master’s or above	2.14	1.11–4.13	0.023*
**History of HIV**			
Yes	1(ref)		
No	0.85	0.72-1.00	0.050
Unsure	0.44	0.30–0.63	< 0.001*

*P < 0.05; AOR means adjusted odds ratio.

## Discussion

In 2022, atypical mpox outbreaks occurred in many non-endemic countries, including the US, Europe, the Middle East, the Western Pacific region, South Korea, and Japan [34, 35]. Thornhill et al. found that 98% of people diagnosed with mpox in 16 countries between April 27 and June 24, 2022 were gay or bisexual men [36]. Because of the connectedness of today’s world, countries must be prepared to face risks in advance. Our study investigates mpox-related knowledge and its associated factors among men who have sex with men in China, aiming to spread knowledge to a targeted population and to take preventive steps to effectively avoid an outbreak of mpox.

This study explores awareness of mpox-related knowledge among Chinese men who have sex with men from five angles: sources of infection, transmission routes, susceptible populations, treatment, and prevention. Only 36.9% of respondents had mpox-related knowledge. Sallam et al. administered an online survey to students at a school of health in Jordan between May 24 and May 26, 2022; they found that the students had poor knowledge of emerging human mpox [37]. Alshahrani et al. reported that more than half (52%) of the general population had an unsatisfactory level of knowledge about mpox infection in Saudi Arabia [38]. These findings suggest that there is a lack of knowledge about mpox. As one of the methods to prevent mpox, it is also necessary to actively publicize the relevant knowledge of mpox to the public. It is particularly crucial to raise awareness of mpox among men who have sex with men as they are a key group.

Significant associations exist between sociodemographic variables and mpox-related knowledge among men who have sex with men. In this study, men who have sex with men who were 33 years and older were more likely to have mpox-related knowledge than those aged 22 and younger. In a study conducted at Al Ain University to evaluate students’ awareness of mpox outbreaks, Jairoun et al. found that older age was a strong determinant of knowledge about mpox in humans [33]. Men who have sex with men can be infected with the mpox virus through close contact during sexual activity [39]. Younger men who have sex with men who have insufficient knowledge of mpox may have a heightened risk of contracting it. Hence, it is important to be aware of increasing preventive interventions and education for younger men who have sex with men. We also found that divorced/widowed men who have sex with men had a higher level of mpox-related knowledge than married individuals. Liu et al. demonstrated that married men who have sex with men are less likely to be exposed to information about the prevention of possible diseases among men who have sex with men [40]. Further, men who have sex with men who are divorced or widowed may receive less support from family members. Compared with married ones, they may more actively seek preventive measures to protect themselves from infection [41]. These results indicate that married men who have sex with men may have a lower level of mpox knowledge, and effective prevention should be targeted at this population. However, due to traditional Chinese culture and attitudes towards gay, men who have sex with men help to conceal their sexual orientation by forming families with women. In this case, different prevention programs need to be offered to married and unmarried men who have sex with men in China.

China’s geography was grouped into the eastern, central, and western regions for our study. Men who have sex with men living in western China may have less knowledge about mpox than those living in the east. This may be because the western region is more rural, with a lower per capita gross domestic product (GDP) and a higher female illiteracy rate than in the eastern and central regions [42]. Education level was significantly associated with mpox-related knowledge. The survey results showed that men who have sex with men with master’s degrees or above had greater awareness of mpox-related knowledge than men who have sex with men with no formal education or primary education only. Consistent with our findings, Wei et al. found that men who have sex with men with higher levels of education had advanced self-awareness against risk [43]. One potential explanation for this outcome is that men who have sex with men with higher education levels may have greater access to information and health literacy, and they can obtain more health knowledge through different information channels. This suggests that mpox-related awareness campaigns need to be expanded to men who have sex with men living in the western parts of China and those who are illiterate or have a primary school education.

Furthermore, sexual health factors contribute to mpox-related knowledge among men who have sex with men. The results indicate that a history of STIs is likely to have an impact on knowledge of mpox among men who have sex with men. Our results demonstrate that people who are unsure of prior HIV infection are less likely to have mpox-related knowledge than those with a history of HIV infection. One possible reason could be that men who have sex with men with a history of HIV received formal sex education at the China Centers for Disease Control and Prevention (CDC), hospital outpatient clinics, or inpatient admissions for diagnosis and treatment, and will take the initiative to pay attention to the diseases that may be associated with men who have sex with men [28]. A growing body of research suggests that men who have sex with men living with HIV use peer education and social networking measures to acquire sexual health-related knowledge [44, 45]. A study reported by Han and colleagues has shown that cooperation with the community of men who have sex with men and community advocacy can promote the sexual health knowledge of men who have sex with men [46]. Fontenot et al. indicated that mobile health interventions are also feasible in promoting sexual health [47]. Men who have sex with men in China frequently use websites, text messages, and mobile apps to search for HIV-related information, counseling, or testing services to reduce HIV-related risk behaviors and improve the effectiveness of care [48, 49]. Furthermore, there may be a link between the increase in STIs, including HIV, and the high prevalence of MPXV outside of Africa; a high proportion of current global mpox cases also involve a history of HIV infection [36, 50]. Studies showed that several initial risk factors for emerging outbreaks of mpox infection, mainly including men who have sex with men, people living with HIV, a history of STIs, including syphilis, and behavioral and health risk factors such as condomless anal intercourse, having multiple sexual partners [51–53]. A study analyzed mpox patients in eight health departments in the United States from May 17 to July 22, 2022, and found that people with HIV infection or sexually transmitted diseases accounted for a high proportion of mpox patients [54]. Cases are more severe following MPXV infection in those who do not realize they are infected with HIV or who are infected but untreated [55]. Overall, because men who have sex with men with a history of HIV may be more susceptible to MPXV infection, individuals living with HIV who have condomless anal intercourse or have multiple sexual partners, but do not know their status, may lack relevant knowledge, and more effective preventive measures (systems for HIV and STI care and prevention) should target this population to prevent the spread of mpox.

However, our findings should be interpreted in light of our study’s limitations. First, the men who have sex with men population is a hardly reached group in China due to different cultural factors. Therefore, this study was conducted by distributing the questionnaire online and obtaining the sample through convenience sampling, which may limit the generalizability to all men who have sex with men in China and produce selection bias. Second, the measures used in the study relied on self-reports, and participants may have had expectations of safe sex (e.g., condom use, number of sexual partners), which could have potential for social desirability bias. Last, incomplete survey items representing mpox awareness and a lack of validated scales for assessment are considerable limitations of this study, which should be considered for future studies.

## Conclusion

This study suggests that mpox-related knowledge is somewhat low among men who have sex with men in China. The factors associated with mpox-related knowledge are age, marital status, region of residence, education level, and a history of HIV. With the current rapid global spread of mpox, public health officials, clinicians, and grassroots health staff need to disseminate knowledge to the public, especially among key groups (e.g., men who have sex with men, HIV-infected individuals, etc.) through multiple channels to increase public awareness and participation before an outbreak occurs. Targeted immunization of groups at high risk of exposure should also be performed to avoid outbreaks.

## Electronic supplementary material

Below is the link to the electronic supplementary material.

**Table S1** Awareness of mpox-related knowledge among MSM in China (N=3,257)

## Data Availability

All data used and analyzed during the current study are available in this article and supplement.

## Abbreviations

MPXV: mpox virus
HIV: human immunodeficiency virus
WHO: World Health Organization
STIs: sexually transmitted infections
DRC: Democratic Republic of the Congo
UK: United Kingdom.
