# Supplementary material for: Awareness of mpox-related knowledge among men who have sex with men in China
Source: BMC Public Health. 2023 Mar 30;23:600. doi: 10.1186/s12889-023-15503-3 (PMC10061892; doi:10.1186/s12889-023-15503-3)
Supplement: Supplementary file 1 — Table S1 Awareness of mpox-related knowledge among MSM in China (N=3,257) [file 12889_2023_15503_MOESM1_ESM.pdf]

Table S1 Awareness of mpox-related knowledge among MSM in China (N=3,257)

Awareness of mpox knowledge	N (%)
**The source of mpox infection**	
Animals infected with mpox	1958(60.1)
People infected with mpox	2238(68.7)
Don't Know	914(28.1)
**The possible transmission routes of mpox**	
Close person-to-person contact	2132(65.5)
Droplet transmission	1293(39.7)
Contact with contaminated objects	1648(50.6)
Vertical transmission	1052(32.3)
sexuality	1737(53.3)
Don't Know	960(29.5)
**Susceptible population**	
All people	1449(35.3)
People who were not vaccinated against smallpox	1235(37.9)
MSM	1123(34.5)
HIV-infected people	941(28.9)
Don't Know	974(29.9)
**Prevention**	
Keep away from areas with mpox outbreaks	2065(63.4)
Get vaccinated	1396(42.9)
Wearing a mask	1560(47.9)
Hand disinfection	1719(52.8)
Regular sex partners and condom use	1698(52.1)
Don't Know	891(27.4)
**Treatment**	
Special drugs to treat	154(4.7)
No specific medicine, symptomatic treatment	1359(41.7)
Don't Know	1744(53.5)
